# Ventralex™ ST Hernia Patch Repair for Small Umbilical Hernia is Safe and Effective: A Retrospective Cohort Study

**DOI:** 10.3389/jaws.2023.11499

**Published:** 2023-06-02

**Authors:** Juha M. Hiekkaranta, Mirella Ahonen, Elisa Mäkäräinen, Pasi Ohtonen, Juha Saarnio, Tero Rautio

**Affiliations:** ^1^ Department of Surgery, Oulu University Hospital, Medical Research Center, University of Oulu, Oulu, Finland; ^2^ Research Service Unit, Oulu University Hospital, The Research Unit of Surgery, Anesthesia and Intensive Care, University of Oulu, Oulu, Finland

**Keywords:** recurrence, umbilical hernia, umbilical hernia repair, ventral patch, hernia patch

## Abstract

**Background:** Hernia patches for umbilical hernia repair have gained popularity due to their short operation time and ease of use. However, up to 10% re-operation and 8% recurrence rates at 2-year follow-up have been published. This retrospective cohort study presents the long-term results of the hernia patch technique for umbilical hernia repair.

**Methods:** All adult patients who underwent a primary umbilical hernia repair at Oulu University Hospital hernia surgery units during 2014–2018 were included in the study. The primary outcome measure was recurrence rate. Secondary outcomes were complications and re-operation rate.

**Results:** A total of 619 elective primary umbilical hernia repairs were performed during 2014–2018. The major technique used was Ventralex™ ST hernia patch repair (79.0%, 488/619) for small hernias with a mean width of 1.8 (SD 0.79) cm. Most of the patches (84.7%, 414/488) were placed in the preperitoneal space. Hernia recurrence rate of patient operated on using Ventralex™ ST hernia patch was 2.5% (12/488) during a mean follow-up time of 68 (SD 16, 43–98) months. Re-operation rate for another reason than recurrence was 1.6% (8/488). Clavien-Dindo complications ≥3 occurred in 4.1% (20/488) of cases and surgical site infection rate was 3.3% (16/488).

**Conclusion:** Umbilical hernia repair using a Ventralex™ ST hernia patch placed in preperitoneal space have acceptable results in terms of recurrence and re-operations in this cohort study.

## Introduction

Umbilical hernia is classified as a primary abdominal wall hernia with no previous surgery to the hernia site located at the midline between the rectus sheaths 3 cm above or 3 cm below the umbilicus [1]. Prevalence of umbilical hernia in adults is about 2% [2]. Umbilical hernia repair is the second most common hernia operation in the Western world [3].

The European Hernia Society (EHS) and American Hernia Society (AHS) recommend the use of a flat permanent mesh in preperitoneal space to repair an umbilical hernia when the hernia defect is larger than 1 cm [4]. Recurrence rate after suture repair of umbilical hernia can be up to 54.5% [2]. Recent meta-analysis concluded that mesh repair compared to suture repair is associated with a lower risk of recurrence and no difference in surgical-site infection, hematomas, or chronic pain [3].

A ventral patch is a preformed mesh designed mainly for small umbilical hernia repairs. The patch can be placed either in intraperitoneal or preperitoneal space through a small fascial defect, after which it self-expands. Hernia repair using a ventral patch is considered a quick and elegant procedure [5]. The composite prosthesis used in Oulu University Hospital, Ventralex™ ST hernia patch (Bard Davol, Warwick, RI, United States), consists of non-absorbable anterior mesh, an absorbable posterior layer and memory ring. The posterior layer is designed to keep the prosthesis from adhering to the intestine [6]. Intraperitoneal behaviour of three composite meshes (Ventralex™ ST hernia Patch, Proceed™ Ventral Patch and Parietex™ Composite Ventral Patch) have been studied on rabbits with serial follow up laparoscopies after implantation. Omental or bowel adhesion were found at 6 weeks in 33%–89% of cases despite different protective layers [7].

Ponten et al. reported significantly more complications and more re-operations in the ventral patch group (Proceed^®^ mesh) compared to preperitoneal flat polypropylene mesh [8]. There are also case reports about mesh migration to bowel after Venralex™ hernia patch used in incisional hernia repair [9]. Several studies have shown low complication, recurrence and reoperation rates after ventral patch repair for small umbilical hernias [5, 6, 10–12]. Nevertheless, authorities from hernia surgical associations have taken a sceptical view of ventral patches, mainly because of costs and complications and recurrences seen after patch repair has been used in improper situations [4]. Most small umbilical hernias in the Oulu University Hospital region have been operated on using Ventralex™ ST hernia patch placed either in preperitoneal or intraperitoneal space. Due to the interest in achieving clarity to this contradiction, the aim of this study is to investigate the long-term results of umbilical hernia patch repair.

## Methods

### Study Design

**FIGURE 1:**
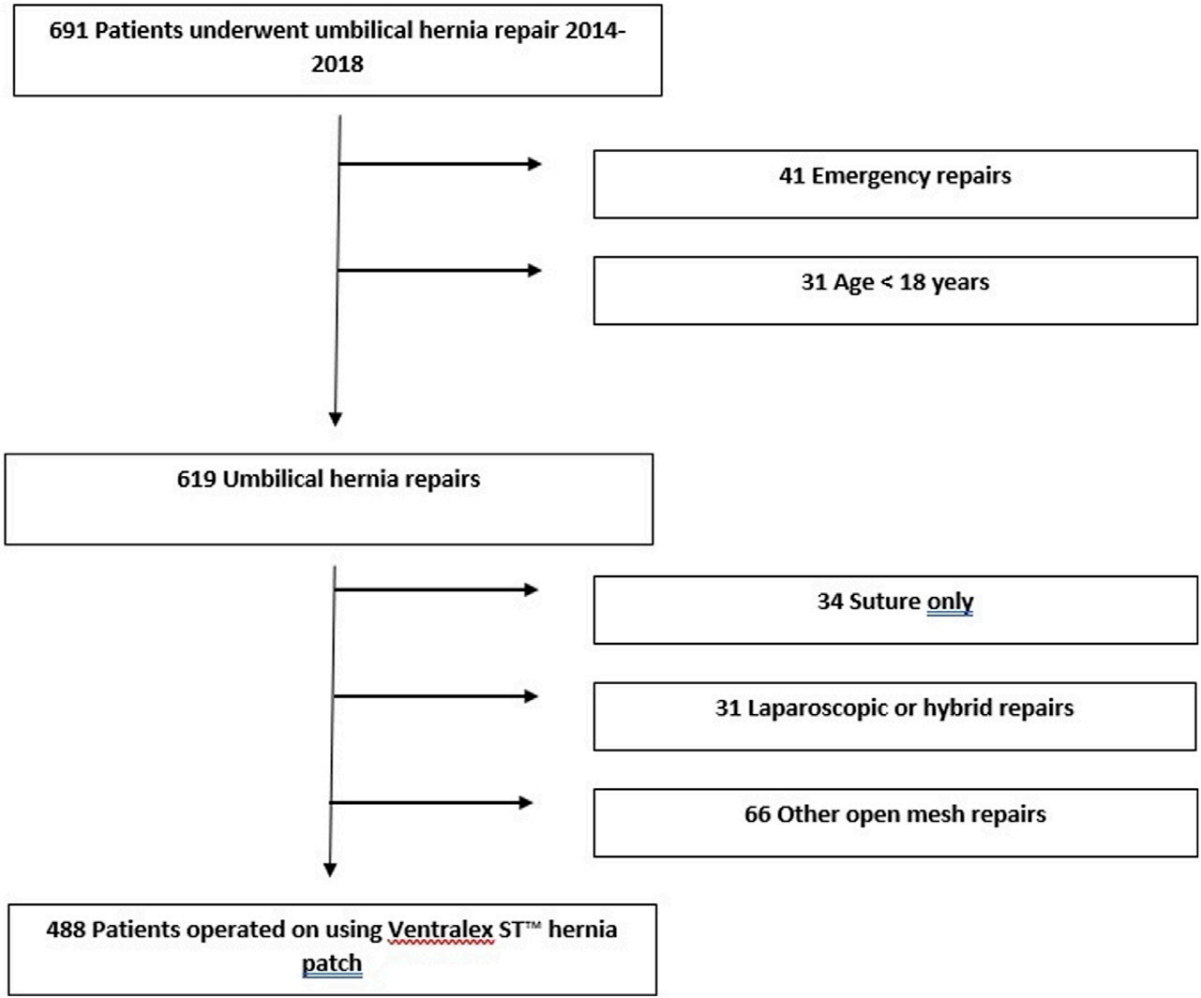
Flow chart of patients in the study.

This is a retrospective cohort study. A surgical database search was conducted to find all patients operated on for primary umbilical hernia in hernia surgery units in Oulu University Hospital between 2014 and 2018. This time period was chosen in order to obtain an adequate follow-up time and number of patients. Exclusion criteria were age under 18, emergency operation, recurrent umbilical hernia repair. Study flow chart is seen in Figure 1. Patients, who had an elective primary umbilical hernia repair using the Ventralex™ ST hernia patch were included. Ethical review board permission was obtained prior starting the study.

Medical records were accessed to find baseline patient information, operative notes, and post-operative course. Follow-up and complication data were collected between January 2022 and May 2022 from patients’ medical files by a single researcher (JH). Number of all operations are presented mainly to describe the entire patient population. Primary endpoints for this study were hernia recurrence and hernia-related re-operations. A patient was considered to have recurrence if recurrent hernia was clinically diagnosed, seen in radiological examination or if patient underwent operation for recurrence. Secondary endpoints were surgical site infection rate (SSI), surgical site occurrence (SSO), other complications graded using Clavien-Dindo classification and chronic pain.

### Statistical Methods

Continuous variables are presented as means with standard deviations (SD), unless otherwise stated. Categorical data are presented as percentages and proportions.

A power analysis was not performed, since all eligible patients during the study period were included in the study. Student’s t-test or Welch’s t-test was used for between group comparisons for continuous variables. The Welch’s t-test was used if the assumption of homogenous variances was not met. Categorical variables were compared using *Χ* ^2^ or Fisher’s exact test. Two-tailed *p*-values are reported. SPSS for windows (IBM Corp. Released 2019. IBM SPSS Statistics for Windows, Version 26.0. Armonk, NY: IBM Corp) was used for statistical analyses.

## Results

Between 2014 and 2018 a total of 619 adult patients underwent elective umbilical hernia repair and 488 of these patients were operated on using the Ventralex™ ST hernia patch (Figure 1). Decisions concerning operative technique were made according to the preference of the operating surgeon.

Baseline of patients and hernia characteristics are presented in Table 1. Two thirds of the patients were males (*n* = 331, 67.9%) with mean age being 49.6 (SD 13.0) years. Patients were relatively healthy with only 11.5% (*n* = 56) having an American Society of Anesthesiologist (ASA) rating of ≥3. The prevalence of smoking was 18% (*n* = 87) at the time of operation. A majority (*n* = 350, 71.6%) of hernias were classified as medium size (1–4 cm) with only 1.6% (*n* = 8) being large (>4 cm). The mean hernia width for Ventralex™ ST hernia patch repair was 1.8 (SD 0.79, 0.3–6) cm. The operating surgeon diagnosed coexistent rectus diastasis in 5.7% (*n* = 28) patients. Patients with noted diastasis had more recurrences 14.3% (*n* = 4) vs. patients with no mention of rectus diastasis 1.7% (*n* = 8) (*p* 0.003).

**TABLE 1:** Description of patient characteristics.

	All patients (*n* = 488)	Recurrence (*n* = 12)	No recurrence (*n* = 476)	*p*-value
Age, mean (SD, range)	49.6 (13.0, 20–88)	43.5 (18.0, 23–77)	49.8 (12.9, 20–88)	0.10
Female, n (%)	157 (32)	7 (58)	150 (32)	0.063
Body Mass Index, kg/m^2^, mean (SD)	28.9 (4.9)	28.8 (6.1)	28.9 (4.9)	>0.9
Comorbidities				
Cardiac disease, n (%)	140 (28.7)	2 (16.7)	138 (29.0)	0.52
Diabetes mellitus, n (%)	58 (11.9)	2 (16.7)	56 (11.8)	0.64
Hepatic disease, n (%)	4 (0.8)	1 (8.3)	3 (0.6)	0.10
Pulmonary disease, n (%)	68 (13.9)	1 (8.3)	67 (14.1)	>0.9
Renal disease, n (%)	4 (0.8)	0 (0)	4 (0.8)	>0.9
Current smoker, n (%)	87 (17.8)	3 (25)	84 (17.6)	0.46
ASA 1, n (%)	178 (36.5)	3 (25)	175 (36.8)	0.60
ASA 2, n (%)	253 (51.8)	7 (58.3)	246 (51.7)	—
ASA 3, n (%)	56 (11.5)	2 (16.7)	54 (11.3)	—
ASA 4, n (%)	1 (0.2)	0 (0)	1 (0.2)	—
Hernia characteristics(*n* = 448)				
Hernia width <1 cm, n (%)	91 (20.3)	3 (30)	88 (20.1)	0.69
Hernia width 1–4 cm, n (%)	349 (77.9)	7 (70)	342 (78.1)	—
Hernia width 4 cm, n (%)	8 (1.8)	0 (0)	8 (1.8)	—
Mean hernia width, cm (SD, range)	1.8 (0.79, 0.3–6)	1.5 (0.39, 1–2)	1.9 (0.8, 0.3–6)	0.12
Rectus diastasis, n (%)	28 (5.7)	4 (33.3)	24 (5.1)	0.003

For continuous variables standard deviation (SD) is presented.

Age (years) at operation is presented in the table.

Operative details for patients operated on with Ventralex™ ST hernia patch are described in Table 2. A majority of cases were performed by experienced surgeons (*n* = 293, 59.9%). Fascial defect was closed in 60 (12.3%) patients. Mesh was placed in preperitoneal space in 414 (84.8%) patients, in intraperitoneal space in 30 (5.9%) patients, and was unclear in 45 (9.2%) patients. The location of the mesh was classified as indistinct if the operating surgeon was not sure that the mesh was completely in the preperitoneal space or if the location remained unclear based on the surgical report. Dissection of the preperitoneal space is usually done through a small hernia port and sometimes it might be difficult to say for sure if there are small tears in the peritoneum or if the small peritoneal tears have been completely closed. A small 4-cm patch was most frequently used (*n* = 228, 46.7%), followed by a medium-sized 6-cm patch (*n* = 177, 36.3%). This resulted in a mean of 1.8 cm (SD 0.63) mesh overlap per side. Most of the operations were performed as a day case surgery with a mean length of stay of 0.7 (SD 0.7, min-max 0–7) days.

**TABLE 2:** Description of operative details in operations using a Ventralex™ ST hernia patch.

	All patients *n* = 488	Recurrence *n* = 12	No recurrence *n* = 476	*p*-value
Specialist operator vs. resident, n (%)	293 (60)	5 (41.7)	288 (60.5)	0.24
Fascia defect completely closed, n (%)	60 (12.3)	4 (33.3)	56 (11.8)	0.048
Antibiotic prophylaxis, n (%)	379/484 (78)	11 (91.7)	368/472 (78.0)	0.48
Hernia completely reducible, n (%)	328/461 (70.9)	9/11 (81.8)	318/450 (70.7)	0.52
Day surgery, n (%)	193 (39.5)	6 (50)	187 (40.7)	0.65
Mesh location, n (%)				0.14
Preperitoneal	414 (84.8)	9 (75)	405 (85.1)	—
Intraperitoneal	29 (5.9)	0 (0)	29 (6.1)	—
Indistinct	45 (9.2)	3 (25)	42 (8.8)	—
Mesh size, n (%)				0.11
4 cm	228 (46.7)	9 (75)	219 (46.6)	—
6 cm	177 (36.3)	3 (25)	174 (37.0)	—
8 cm	77 (15.8)	0 (0)	77 (16.4)	—
Mesh overlap cm, mean (SD, min–max)	1.8 (0.63, 0.5–3.8)	1.5 (0.42, 1–2.3)	1.8 (0.64, 0.5–3.8)	0.13
<1 cm, n (%)	69/442 (15.6)	2/10 (20)	67/432 (15.5)	0.87
1–2 cm, n (%)	237/442 (53.6)	6/10 (60)	231/432 (53.5)	—
2–3 cm, n (%)	125/442 (28.3)	2/10 (20)	123/432 (28.5)	—
≥3 cm, n (%)	11/442 (2.5)	0/10 (0)	11/432 (2.5)	—
Suture material to fixate the mesh				0.61
Fast absorbable, n (%)	11/458 (2.4)	0 (0)	12/470 (2.3)	—
Slow absorbable, n (%)	8/458 (1.7)	0 (0)	8/470 (1.7)	—
Non-absorbable, n (%)	438/458 (95.6)	12 (100)	438/470 (95.7)	—

Mesh overlap is reported in cm per side.

Long-term results for Ventralex™ ST hernia patch repair are summarized in Table 3. Hernia recurrence was found in 12 (2.5%) patients during the mean follow-up time of 68 (SD 16) months. Reoperation rate from any cause was 4.1% (*n* = 20). Three of the patients underwent reoperation due to fistula, three for chronic pain and rectus diastasis (two of these patients also had recurrence) and another three also for pain and excess scar formation or abnormal reaction to mesh. One patient was operated on for SSI. Fistulas were skin to mesh or suture fistulas. No enterocutaneous fistulas was found in this series. In reoperations due to fistula mesh was not explanted. Mesh was removed in seven patients during reoperations for chronic pain or infection or recurrence. Preperitoneal mesh location resulted 7.7% (*n* = 32), intraperitoneal 10.3% (*n* = 3) and Indistinct 17.8% (*n* = 8) Claviend-Dindo ≥2 complication rate (*p* 0.041). Recurrence rate with preperitoneal mesh was 2.2% (*n* = 9), intraperitoneal 0% (*n* = 0) and with indistinct 6.7% (*n* = 3) (*p* 0.12). Similarly Clavien-Dindo ≥3 complication rates were 3.6% (*n* = 15) with preperitoneal mesh, 0% (*n* = 0) intraperitoneal and 11.1% (*n* = 5) for indistinct (*p* 0.05). All three patients operated for chronic pain were originally operated with fascial defect left open. Symptomatic seromas also occurred in patients whose fascial defect was not closed. Patient characteristics (Table 1) and operation data (Table 2) are also presented separately for patients with recurrence and for patients without recurrence.

**TABLE 3:** Description of results for patients operated on using Ventralex ST™ hernia patch.

	All patients (*n* = 488)	Recurrence (*n* = 12)	No recurrence (*n* = 476)	*p*-value
Hernia recurrence, n (%)	12 (2.5)	—	—	
Hernia recurrence operated, n (%)	12 (2.5)	—	—	
Re-operation for other reason, n (%)	8 (1.6)	3 (33)	5 (2.9)	
SSI, n (%)	16 (3.3)	0	16 (3.3)	>0.9
Seroma, n (%)	3 (0.6)	0	3 (0.6)	>0.9
Hematoma, n (%)	5 (1.0)	0	5 (1.1)	>0.9
SSO, n (%)	40 (8.2)	—	—	
SSOPI, n (%)	20 (4.1)	—	—	
Chronic pain, n (%)	8 (1.6)	1 (8.3)	7 (1.5)	0.18
Clavien-Dindo, during follow-up time, n (%)				—
1	2 (0.4)	—	—	
2	23 (4.7)	—	—	
3	20 (4.1)	—	—	
30-day Clavien-Dindo, n (%)				—
1	2 (0.4)	—	—	
2	26 (5.7)	—	—	
3	2 (0.4)	—	—	
Follow-up time months, mean (SD, min-max)	68 (16, 43–98)	72 (15, 48–93)	68 (16, 43–98)	0.32

Standard deviation (SD) is presented for continuous variables.

Surgical site occurrence (SS0) includes seromas and hematomas.

Surgical site occurrences requiring procedural interventions (SSOPI).

## Discussion

In line with previous reports, this study showed that the use of the Ventralex™ ST hernia patch placed in preperitoneal space in umbilical hernia repair has acceptable results in terms of recurrence and complications.

Porrero et al. [6] reported similar complication rates in their retrospective series. Studies with smaller numbers of patients have had more mixed results, with recurrence rates ranging from 0% to 8.9% [5, 11, 13, 14]. To our knowledge, there is only one published randomized controlled trial comparing flat preperitoneal polypropylene mesh with an intraperitoneal or preperitoneal Proceed^®^ ventral patch in small umbilical hernia repairs. In this study, significantly more complications and more re-operations in the ventral patch group were reported compared to flat mesh [8].

European and American hernia associations recommend in their guidelines that symptomatic umbilical hernias should be repaired using an open approach with a preperitoneal flat mesh. In the associations’ guideline article, the authors concluded that the use of intraperitoneal preformed patches for umbilical hernia repairs may shorten operating time, but may be associated with increased complication rates compared with placing a flat mesh in the preperitoneal space. They also point out the high costs of pre-shaped prosthetics with anti-adhesive barriers [4].

There are several different techniques for mesh placement in the preperitoneal space using open surgery, laparoscopic or robotic approaches. For smaller umbilical hernias, open technique using ventral patches is attractive because the procedure is quick and simple even for less-experienced surgeons. There are numerous composite hernia patches on the market, and these can in most cases be inserted into preperitoneal or intraperitoneal space. The significance of patch placement either in preperitoneal or intraperitoneal space remains unclear [8]. Intraperitoneal Ventralex™ hernia patch has shown similar complication rates but lower early postoperative pain scores when compared to onlay mesh [11]. The most severe complications are related to mesh bowel attachment or abdominal mesh migration favouring preperitoneal mesh placement. It is not easy to avoid peritoneal tears during the preperitoneal space dissection or mesh placement pass relatively small hernia port. Because of this it might be beneficial to use mesh with antiadhesive barrier even when mesh is placed preperitoneally. Preperitoneal technique secure adequate mesh contact to abdominal wall, whereas in intraperitoneal mesh placement preperitoneal fat might interfere mesh integration. In this cohort preperitoneal location of the mesh decrease recurrence and complication rates compared to intraperitoneal or indistinct mesh locations. However, the study design was not intentional for this kind of comparison and our results are not all statistically significant. Superiority of preperitoneal placement of Ventralex™ has not been proven in comparative study [15].

In this study population, almost one-fifth of patients had a small hernia (width<1 cm) where mesh repair was not recommended. However, there is recent evidence favouring mesh repair also in this patient group. A Danish register study concluded that mesh reduces recurrences even in small hernias but increases early complications. In this series onlay mesh repairs had a lower recurrence rate compared to other types of meshes [16].

In this study population, fascia defect was mostly left open and mesh was sutured to the edges of the defect, contrary to recent guideline recommendation by EHS. Fascial defect closure is one of the essential part of all types of ventral hernia repair techniques. It has been shown to reduce hernia recurrences and SSO in laparoscopic intraperitoneal onlay mesh repairs [17, 18]. Leaving the fascia open did not seem to cause a large number of recurrences in this study. However, mesh to skin or suture fistulas and mesh reactions led to several (6) re-operations. These complications might be avoidable if the fascia is closed. Therefore, we recommend that the fascia defect should be closed when using a preformed hernia patch. A large proportion of patients received small (4 cm) mesh, although current guidelines recommend a minimum of a 2-cm mesh overlap which is impossible to achieve with small mesh. Mesh overlap of less than 1 cm has shown to be a risk factor for recurrence when using Ventralex™ ST hernia patch for umbilical hernia repair [19]. Although the mesh overlap is smaller than recommended, again it did not seem to cause problems in this series when median hernia size was relatively small.

## Limitations

This is a cohort study with all limitations in relation to retrospective data collection. A major problem is caused by the fact that surgical reports for pre- and intraoperative parameters are incomplete. Surgical technique was not standardized, and surgeons used what they were familiar with or what they thought was best for the given patient (e.g., whether to close the defect or not, or what size mesh to use), which led to heterogeneity. Further, follow-up data were collected merely from the medical records, which inevitably led to underreporting of complications.

Still, we believe that recurrence rate and (Clavien-Dindo ≥3) complications are close to correct since data were collected from our own hernia surgical units within our geographically vast hospital district. Patients usually seek treatment for complications at the hospital where they were operated on.

Information bias may exist depending on surgeons’ choice of operative method and the differing quality of individual operative reports. Simple hernia surgery in Finland is done by general surgeons who have adopted the patch repair technique since Ventralex™ ST hernia patch has been available. Hereby, a majority of surgeons were experienced and familiar with the technique. However, our patient cohort is highly selected, and results might not be generalizable. It should be noted that patients in this cohort were relatively healthy in terms of comorbidities and mostly of working age. Despite the retrospective nature of this study, the outcomes reflect normal clinical practice in our hospital. Our study design did not give us the possibility to make any kind of comparative analysis of different operative techniques or meshes.

One of the major limitations is that we do not have any data on patient satisfaction or quality of life, which should be explored in future studies on umbilical hernia repair. According to previous study patients reported mostly good or excellent satisfaction 2 years after umbilical or incisional hernia repair using Ventralex™ ST hernia patch [20].

Risk factor analysis for recurrence is impossible due to the small number of recurrences. Based on the results of this cohort, the hernia patch is a feasible method to repair a small primary umbilical hernia. The preferred location of the mesh cannot be stated based on the results of this cohort. Finally, for specific patient groups, such as patients with an umbilical hernia larger than 4 cm, patients with rectus diastasis or overweight patients, other surgical options should be considered as well.

## Data Availability

The raw data supporting the conclusion of this article will be made available by the authors, without undue reservation.

## Abbreviations

AHS, American hernia society; ASA, American society of anesthesiologists; BMI, body mass index; EHS, European hernia society; SD, standard deviation; SSI, surgical site infection; SSO, surgical site occurrence; SSOPI, surgical site occurrence requiring intervention.
